# Ex situ arterial reconstruction prior normothermic machine perfusion of liver grafts

**DOI:** 10.1007/s00423-022-02611-8

**Published:** 2022-07-20

**Authors:** Felix Becker, Felicia Kneifel, Arne Riegel, Shadi Katou, Tristan Wagner, Isabelle Flammang, Mazen Juratli, Thomas Vogel, Sonia Radunz, Haluk Morgul, Andreas Pascher, Philipp Houben, Jens G. Brockmann

**Affiliations:** 1 Department of General, Visceral and Transplant Surgery, University Hospital Münster, Waldeyerstrasse 1, 48149 Münster, Germany; 2 Clinic for Radiology, University Hospital Münster, Münster, Germany

**Keywords:** Normothermic machine perfusion, Arterial anatomy, Hepatic artery, Liver transplant

## Abstract

**Purpose:**

Atypical variants of the hepatic artery are common and pose a technical challenge for normothermic machine perfusion (NMP). The transplant surgeon has three options when confronted with hepatic arterial variation in a liver graft to be subjected to NMP: to perform arterial reconstruction (i) prior, (ii) during, or (iii) following NMP.

**Methods:**

Herein, we report our experience and technical considerations with pre-NMP reconstruction. Out of 52 livers, 9 had an atypical hepatic artery (HA): 3 replaced right HA, 3 replaced left HA, 1 accessory left HA, 1 accessory left and right HA, and 1 replaced left and right HA.

**Results:**

Reconstruction was conducted during back-table preparation. A single vascular conduit was created in all grafts to allow single arterial cannulation for NMP, necessitating only one arterial anastomosis within the recipient. All grafts were subjected to NMP and subsequently successfully transplanted.

**Conclusion:**

Our approach is being advocated for as it preserves the ability to alter the reconstruction in case of problems resulting from the reconstruction itself, thereby allowing functional evaluation of the reconstruction prior transplantation, permitting simultaneous reperfusion in the recipient, and providing the shortest possible duration for vascular reconstruction once the graft is rewarming non-perfused within the recipient. In addition, in light of the frequency of technically demanding reconstructions with very small vessels, we consider our technique beneficial as the procedure can be performed under ideal conditions at the back-table.

## Introduction

Normothermic machine perfusion (NMP) is a rapidly evolving technology in the field of liver transplants, allowing preservation and assessment of grafts prior to transplantation [1]. Currently, two randomized controlled trials have demonstrated a reduction in post-operative graft injury as well as ischemia–reperfusion injury following NMP [2, 3]. The rapid implementation of NMP in various centers is humbled by the ongoing debate whether atypical variants of the hepatic artery (HA) are a potential contraindication for NMP. There is an urgent and unmet clinical need to close this gap of knowledge, as these grafts have been declined by centers using NMP. Of interest, even in the first randomized controlled trial on NMP, one liver was randomized to NMP but, subsequently, cold-stored due to an accessory left HA preventing effective cannulation in that particular case.

Arterial perfusion is ideally conducted via single vessel cannulation, and thus, atypical arterial anatomy poses a technical challenge for NMP. Additionally, it is preferable to perform only one singular arterial anastomosis in the recipient, instead of having several separate arterial anastomoses in-between donor and recipient vessels. The transplant surgeon has three options when confronted with hepatic arterial variation in a liver graft to be subjected to NMP: to perform arterial reconstruction (i) prior, (ii) during, or (iii) following NMP. The latter is to be done on a back-table or the recipient with the graft in situ. Pre-NMP reconstruction will prolong cold ischemia time (CIT); reconstruction during NMP will impair first arterial reperfusion; while post-NMP reconstruction will prolong the time for proper arterial perfusion in the recipient and may be more difficult as the graft is already in the recipient. Nevertheless, this option may be valuable for perfect trimming, especially with respect to arterial length.

Although aberrant arterial anatomy is frequent [4] and presents in approximately 30% of all liver grafts [5], there is a scarcity of data regarding arterial reconstruction prior NMP and the performance of liver grafts with reconstructed arterial anatomy during NMP. Being one of the leading centers for liver NMP in Europe, we herein report nine cases of HA reconstruction prior NMP, derived from the first 52 NMPs performed at the University Hospital Münster from 10/2019 until 4/2021, and share our experience and technical considerations. This approach is being advocated as it preserves the ability to alter the reconstruction in case of problems resulting from the reconstruction itself, thereby allowing functional evaluation of the reconstruction prior to transplantation, permitting simultaneous reperfusion in the recipient, and providing the shortest possible duration for vascular reconstruction once the graft is rewarming non-perfused within the recipient. In addition, in light of the frequency of technically demanding reconstructions with very small vessels, we consider our technique beneficial as the procedure can be performed under ideal conditions at a back-table.

## Material and methods

### Study design

We retrospectively reviewed our prospectively collected NMP database for cases between October 2019 and April 2021 at the Department of General, Visceral and Transplant Surgery, University Hospital Münster, Germany. The study was conducted according to the ethical principles of the Declaration of Helsinki. The approval of the local Ethics Committee was obtained (Ethik-Kommission der Ärztekammer Westfalen-Lippe und Westfälischen Wilhelms-Universität, No. 2019–673-f-S). Prior to the analysis, all data were de-identified, and all participating patients provided their written consent for the routine recording of clinical data.

**Fig. 1:**
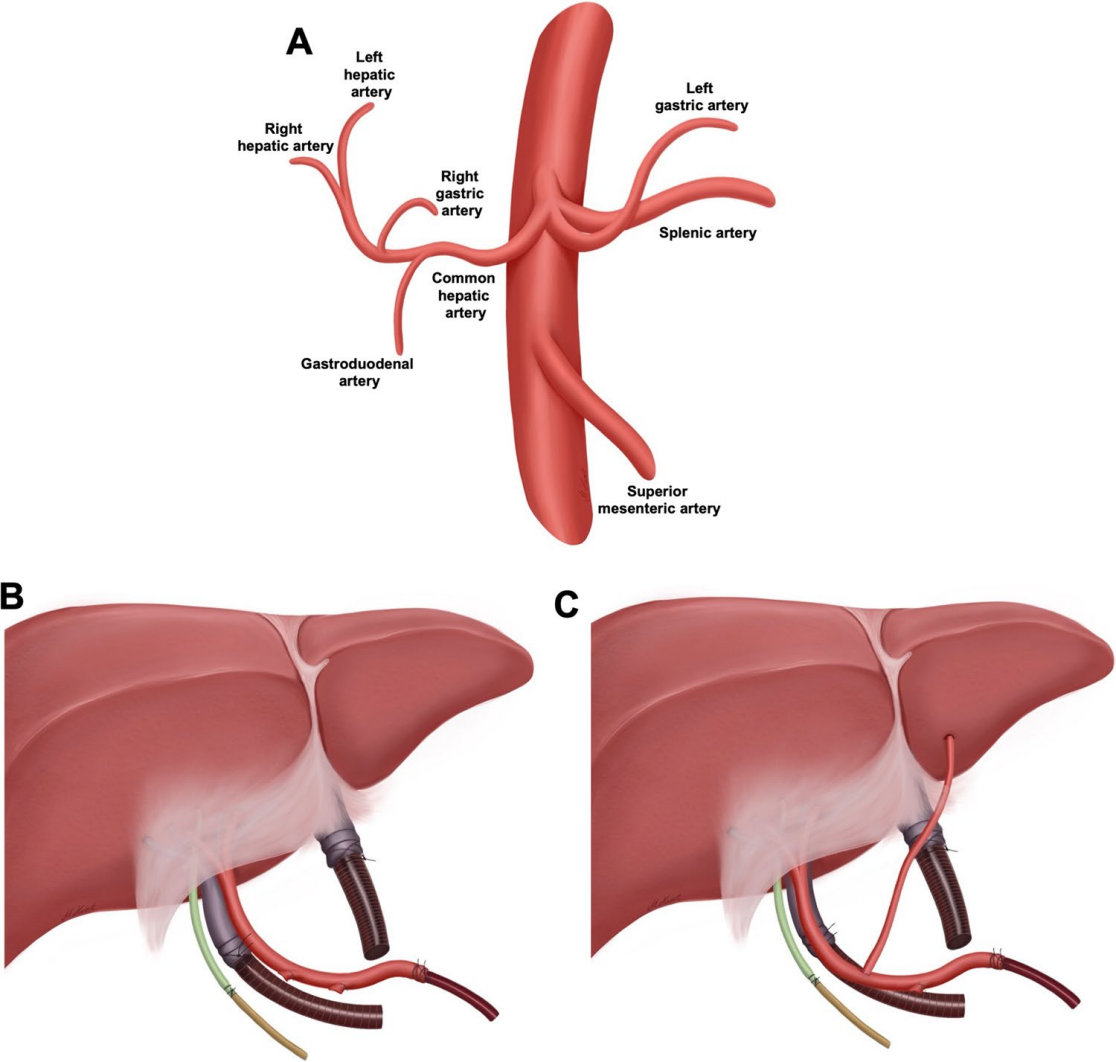
Regular anatomy of the celiac trunk and cannulation for normothermic machine perfusion. (**A**) Regular anatomy of the celiac trunk and proximal superior mesenteric artery. (**B**) Standard cannulation technique of a liver graft prior normothermic machine perfusion with a single cannulation of the bile duct, portal vein, hepatic artery, and vena cava. (**C**) Example of a single cannulation following the arterial reconstruction of an accessory left hepatic artery

The inclusion criterion was a full liver allograft with any form of anatomical HA variation. Standard hepatic arterial anatomy (type I according to Michel’s classification) was defined as a single common hepatic artery (CHA) proceeding from the celiac trunk, giving rise to the gastroduodenal artery (GDA), right gastric artery (RGA), and lastly, proper left (LHA) and right (RHA) HA (Fig. 1A**)**. Aberrant HA anatomy was categorized according to Michels’ [6] and Hiatt’s [7] classification. Accessory (a) arteries were defined as occurring in addition to the normal arterial pattern and a replaced (r) artery was defined as a variant that was the primary arterial supply for the respective left or right lobe. At the time of allocation, aberrant HA anatomy was known for six grafts (66.6%), while variations in the remaining grafts were discovered later, during back-table preparation. Thus, the HA anatomy had no specific role in the allocation process, as even complex reconstructions were not defined as a contraindication for NMP.

### Arterial reconstruction

Surgical reconstruction of the arterial tree followed two main principles in all cases: (i) creating a single vascular conduit enabling single vessel cannulation for NMP (and aiming for only one single anastomosis in the recipient) and (ii) mandatory reconstruction of all vessels, irrespective of whether accessory or replaced.

Reconstruction was performed prior NMP, during back-table preparation using microvascular techniques and surgical loupes. The utilization of an operating microscope was never necessary. Prior to reconstruction, the aortic Carrel patch encompassing the origin of both the superior mesenteric artery (SMA, when applicable) and the celiac trunk with its branches were meticulously dissected, enabling a clear classification of variants. Reconstruction was mainly conducted with a continuous 7–0 or 8–0 Prolene suture. For lumina, smaller than 3 mm interrupted sutures were applied. Using temporary stenting (5F irrigation cannula) across the anastomotic site facilitated secure suture placement. The vascular anastomosis was mainly end-to-side. An end-to-end reconstruction onto the GDA was performed in one case. To avoid any form of kinking, the length of reconstructed vessels was kept to an absolute minimum, and all anastomoses were conducted at the origin of the GDA or distal to it. Thus, accessory hepatic arteries (aHAs) were reconstructed using the origin of the GDA or RGA, while replaced hepatic arteries (rHAs) were reconstructed directly on the respective HA arising from the celiac trunk.

#### NMP

Post-static cold storage NMP, as defined by Karangwa et al. [8], was conducted using the OrganOx metra device [2] with a back-to-base protocol. In this process, liver grafts are procured, transported to the transplant center, and subsequently connected to an NMP device. Following standard back-table preparation with skeletonization of the inferior vena cava (IVC), portal vein (to the level of its bifurcation) and HA, each vessel was cannulated with a single cannula. First, the suprahepatic IVC was closed with a running suture and the infrahepatic IVC was cannulated. Next, the portal vein was cannulated, followed by the cannulation of the reconstructed HA at the origin of the celiac trunk using a 10 F tube. Then, all the remaining branches (e.g., GDA, RGA, left gastric artery (LGA), and splenic artery (SA)) were tied off. Lastly, the bile duct was cannulated, and the liver was connected to the OrganOx metra device (Fig. 1B, C). Following the start of perfusion, the anastomotic patency was controlled manually through palpation. To evaluate metabolic graft function, lactate was tested 15 min after beginning perfusion and hourly thereafter.

### Liver transplant and follow-up

Graft implantation was performed using the modified piggyback technique (Belghiti) with end-to-side cavo-caval anastomosis and temporary portocaval shunting. The arterial anastomosis was performed using end-to-end anastomosis at the level of the bifurcation of the proper HA or GDA. Intraoperative patency of the reconstructed arterial tree as well as the main anastomosis (graft-to-recipient) was confirmed through Doppler ultrasound (US) and intraoperative flow measurements.

Our post-operative standard procedure included Doppler US following skin closure and every 12 h within the first 5 days, as well as once daily afterward; no other post-operative imaging was obtained without indication. However, all patients received a CT scan within the first year of transplant (66 ± 165 days). The use of anticoagulation was not determined by arterial reconstruction, and patients received anti-platelet therapy only when indicated by the leading transplant surgeon.

### Statistics

All data were analyzed with Graph Pad Prism (Version 9, Graph Pad Software, San Diego, CA, USA). Continuous variables are presented as mean values ± standard deviation (SD), and statistical significance between groups was analyzed using the unpaired Student’s *t*-test. For categorical variables, the Fisher’s exact test was used. One-year patient survival, death-censored graft, and overall graft survival were estimated by Kaplan–Meier methodology. A value of *p* < 0.05 was considered statistically significant.

## Results

**Fig. 2:**
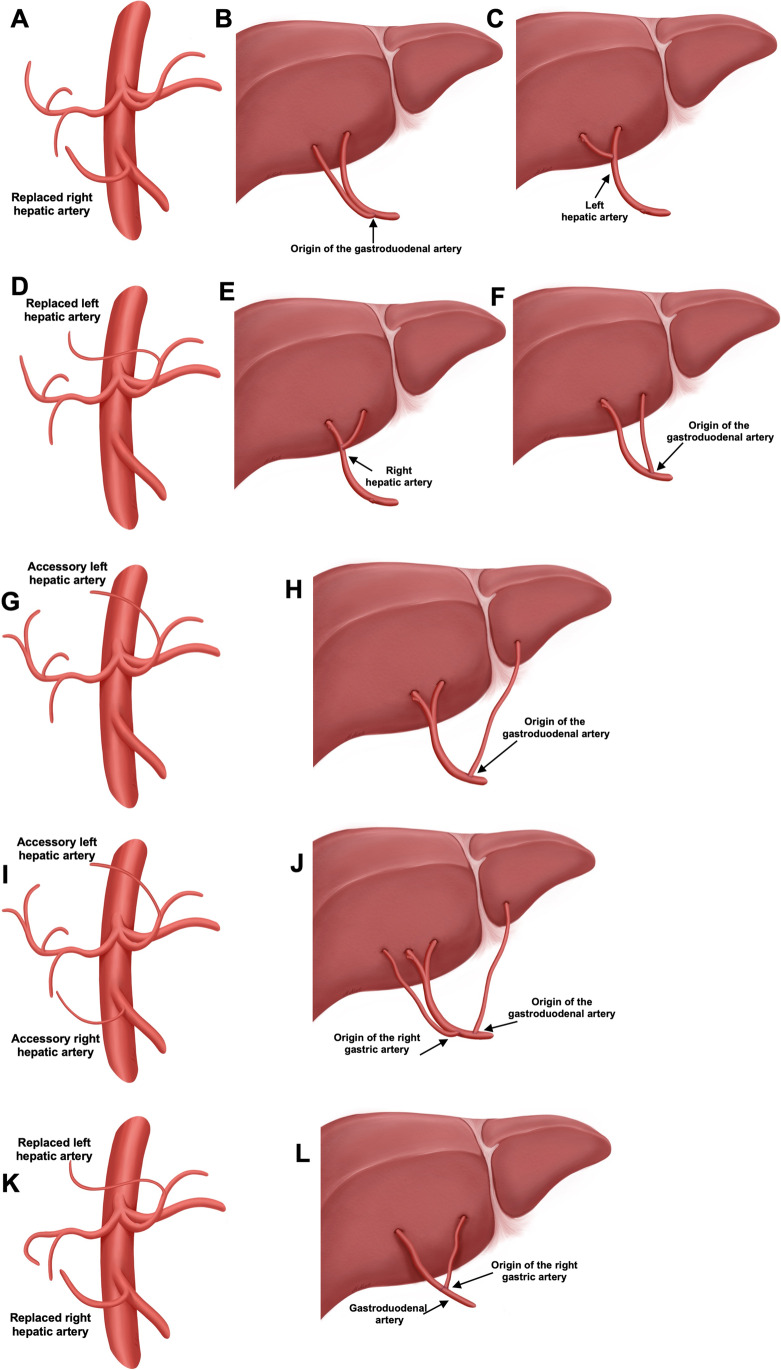
Variations of the hepatic artery and reconstruction. Nine liver grafts (18%) displayed an anatomical variation of the hepatic artery. Three grafts displayed a replaced right hepatic artery (**A**), which was reconstructed to the origin of the gastroduodenal artery (*n* = 2, **B**) or directly to the left hepatic artery (*n* = 1, **C**). Three grafts displayed a replaced left hepatic artery (**D**), which was reconstructed directly to the right hepatic artery (*n* = 2, **E**) or the origin of the gastroduodenal artery (*n* = 1, **F**). One graft displayed an accessory left hepatic artery (**G**), which was reconstructed to the origin of the gastroduodenal artery (**H**). One graft displayed an accessory left hepatic artery as well as an accessory right hepatic artery (**I**); while the right artery was reconstructed to the origin of the right gastric artery, the left was reconstructed to the origin of the gastroduodenal artery (**J**). One graft displayed a replaced left and a replaced right hepatic artery (**K**). The right was reconstructed to the gastroduodenal artery and the left was reconstructed to the origin of the right gastric artery

Between October 2019 and April 2021, a total of 52 livers (recipient and donor details are listed in Table 1) were perfused using the OrganOx metra device, of which nine livers (18%) displayed HA variations: in three cases, an rRHA from the SMA (Michel’s type 3, Fig. 2A) was found and reconstructed by using the origin of the GDA (Fig. 2B) to combine it with the CHA or by directly performing an anastomosis of the rRHA to the LHA (Fig. 2C). We observed three cases of an rLHA arising from the LGA (Michels’ type 2, Fig. 2D). These were reconstructed either directly onto the RHA (Fig. 2E) or by using the origin of the RGA (Fig. 2F). One graft had an aLHA from the LGA (Michels’ type 5, Fig. 2G), which was reconstructed to the CHA at the origin of the GDA (Fig. 2H). Another graft displayed an aLHA (from the LGA) and an aRHA (from the SMA) (Michels’ type 7, Fig. 2I). Both arteries were reconstructed to the CHA by using the origin of the GDA (for the aLHA) and RGA (for the aRHA). The third graft displayed both rLHA as well as rRHA (Fig. 2K**)**; while this variant is not included in Michel’s classification, it was later included in the simplified Hiatt classification (Hiatt’s type IV). As this variant is characterized by the absence of a CHA and a GDA arising directly from the celiac trunk, the rRHA was reconstructed end-to-end to the GDA, while the rLHA was reconstructed end-to-site to the origin of the RGA (Fig. 2L).

**Table 1:** Recipient and donor characteristics

Recipient characteristics	
Age (years, mean ± SD)	54 ± 13
Gender (% males)	44
Indication for transplant (*n*, (%))	
ITBL	1 (11)
HCC	2 (22)
PBC	1 (11)
Viral hepatitis	1 (11)
SSC	2 (22)
DILI	1 (11)
Alcoholic cirrhosis	1 (11)
MELD (mean ± SD)	23 ± 10
Donor characteristics	
Age (years, mean ± SD)	54 ± 8
Gender (% males)	33
BMI (kg/m^2^, median)	27 ± 5
DRI (mean ± SD)	1.76 ± 0.22

*ITBL* ischemic type biliary lesions, *HCC* hepatocellular carcinoma, *PBC* primary biliary cirrhosis, *SSC* secondary sclerosing cholangitis, *DILI* drug-induced liver injury, *MELD* model for end-stage liver disease, *BMI* body mass index, *DRI* donor risk index

**Fig. 3:**
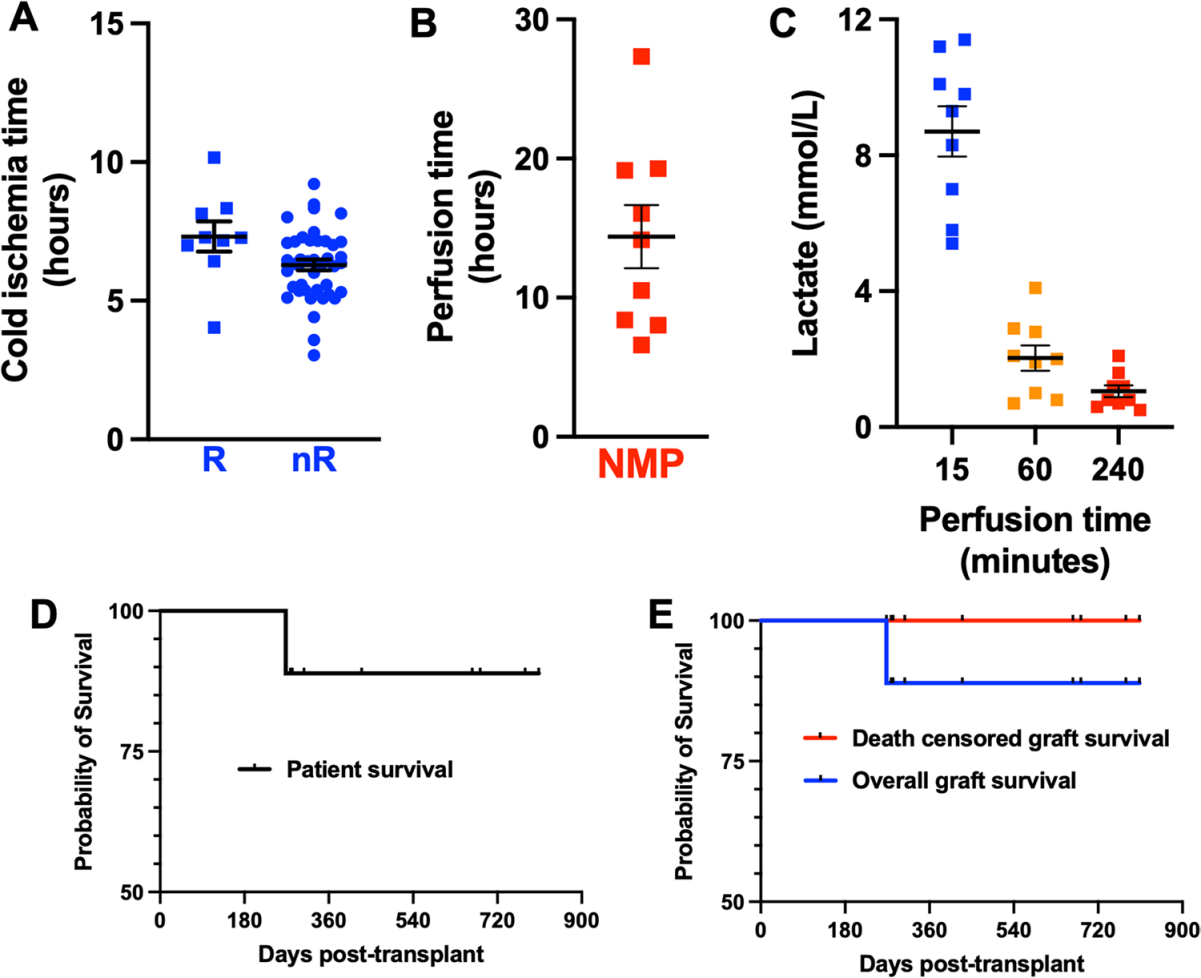
Graft performance during normothermic machine perfusion and post-transplant outcome. (**A**) Cold ischemia time (CIT) of livers with reconstruction (*R*, *n* = 9) and without reconstruction (nR, *n* = 43). (**B**) Duration of normothermic machine perfusion (NMP) of livers with arterial reconstruction. (**C, D**) Lactate clearance during NMP after 15, 60, and 240 min of perfusion. (**E**) Patient survival following a liver transplant. (**F**) Overall (blue) and death censored (red) graft survival following a liver transplant

Livers were perfused following a CIT of 7.3 ± 1.6 h. Of interest, the CIT was comparable to that of the 43 livers without arterial reconstruction (6.3 ± 1.2 h) subjected to NMP during the study period (Fig. 3A). Although we have no exact data regarding the time needed for reconstruction, it never exceeded 30 min, and differences in CIT are most likely due to logistics. Reconstructed grafts were kept on the OrganOx metra device for 14.4 ± 6.8 h (Fig. 3B). All grafts were perfused normally and showed a homogenous color and regular macroscopic appearance, which was maintained throughout perfusion. Directly after starting NMP, the anastomotic patency was confirmed manually and, in case of doubt, flow measurement and Doppler US were carried out. All grafts showed sufficient lactate clearance with a drop from 8.7 ± 2.2 mmol/l (at 15 min) to 2 ± 1.1 mmol/l (at 60 min) to subsequently 1.1 ± 0.5 mmol/l (at 240 min, Fig. 3C). All nine reconstructed grafts achieved regular HA flows (422 ± 132 ml/min at 1 h) and remained stable during the perfusion period (Fig. 3C). At the end of perfusion, no sign of HA thrombosis or stenosis was present.

To analyze whether arterial reconstruction prior NMP had any effect on the perfusion itself, we assessed functional metabolic as well as flow parameters (after 4 h of perfusion) between the nine reconstructed grafts and 43 livers without arterial reconstruction subjected to NMP during the study period. No differences were found with respect to weight-adjusted HA flow (with reconstruction: 0.27 ± 0.03 ml/g/min; without reconstruction: 0.30 ± 0.1 ml/g/min; *p* = 0.374), weight-adjusted portal vein flow (with reconstruction: 0.74 ± 0.13 ml/g/min; without reconstruction: 0.7 ± 0.16 ml/g/min; *p* = 0.583), lactate concentration (with reconstruction: 1.05 ± 0.52 mmol/l; without reconstruction: 1.19 ± 0.67 mmol/l; *p* = 0.587) or pH (with reconstruction: 7.18 ± 0.08; without reconstruction: 7.22 ± 0.11; *p* = 0.395).

**Fig. 4:**
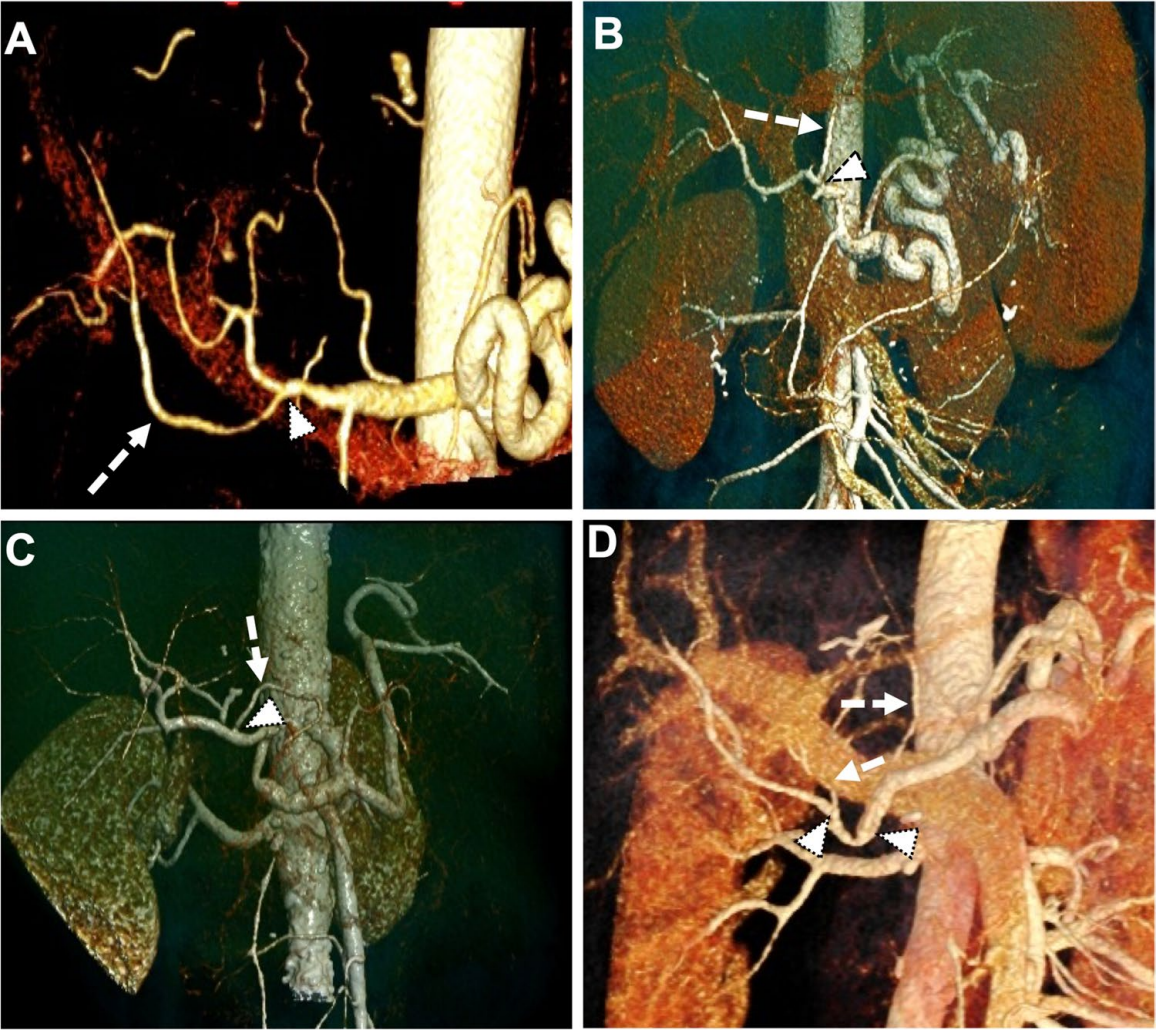
Computed tomography with selective reconstruction of the vascular anatomy. Representative images from 4 scans showing a reconstructed (**A**) replaced right hepatic artery, (**B**) replaced left hepatic artery, (**C**) accessory left hepatic artery, and (**D**) accessory left and right hepatic artery. White arrowheads with black lining indicate the site of reconstruction, and white arrows indicate the reconstructed vessel

All grafts were successfully transplanted, and simultaneous reperfusion was carried out in six cases (67%). In three cases, hemodynamic instability of the recipients necessitated primary portal vein reperfusion. Intraoperative Doppler US and flow measurements revealed regular values, and no HA thrombosis or bleeding at the site of reconstruction was observed. Following reperfusion in the recipient, no additional reconstruction or altering of the pre-NMP reconstruction was necessary. No anastomosis needed to be redone or refashioned. A total of six patients (67%) received post-operative platelet anti-aggregating, which was continued for the first six post-surgical weeks. Post-operative Doppler US conducted during the first 5 days revealed a regular (0.5–0.8) arterial resistive index (RI; 0.67 ± 0.08) in all cases but one. One patient presented with a high-resistance flow with a normal systolic phase and a continuous but decreased diastolic flow (RI 0.86) on post-operative day 2 in a singular measurement. However, all subsequent examinations showed normal indices and no further intervention was required. None of the cases developed late HA thrombosis or stenosis. All cases demonstrated full arterial patency proven by post-operative CT scans. Figure 4 displays four representative examples of a right rHA (Fig. 4A), a left rHA (Fig. 4B), a left aHA (Fig. 4C) and left and right aHA (Fig. 4D).

Frequency of early allograft dysfunction [9] within the first 7 post-operative days was 33.3%, which was comparable to grafts without reconstruction (39.5%; *p* = 0.728). The 90-day graft and patient survival was 100% (Fig. 3D), while 1-year patient survival was 89% (Fig. 3E) because of one death with a functional graft at day 269 post-transplant due to a catheter-related sepsis in a previously heart-transplanted patient with chronic kidney failure. Mean follow-up was 500 ± 218 days. All eight surviving recipients are well at the end of follow-up.

## Discussion

Our data provides evidence that atypical HA anatomy is not a contraindication for NMP. Arterial reconstruction prior to NMP is safe and feasible. Our report provides technical details of pre-NMP reconstruction of the hepatic arterial tree as well as the largest case-series regarding functional NMP parameters of reconstructed liver grafts. The aim of this study was to contribute to the rapidly evolving implementation of NMP, and our data on the technical feasibility of pre-NMP reconstruction should advice the transplant community that no graft should be deemed unsuitable for NMP based on arterial anatomy.

Since HA integrity is key for successful liver transplantation, we created a straightforward and progressive approach of pre-NMP arterial reconstruction resulting in a single vascular conduit enabling singular vessel cannulation and the necessity for only one arterial anastomosis within the recipient. The pre-NMP arterial reconstruction approach has several advantages when compared to reconstruction during or following NMP. First, when compared to reconstruction during NMP, our approach allows reconstruction without compromising graft perfusion in the most comfortable exposure during back-table preparation. Especially when a large rHa is reconstructed during NMP (after an initial dual canulation), one must consider that during the time of reconstruction, not only is the respective replaced artery closed but it may also be required to temporarily close the remaining proper HA, creating an additional phase of warm ischemia without arterial inflow. Additionally, reconstruction of very small and fragile accessory arteries maybe time consuming, which also subjects the graft to warm ischemia. However, the impact of a temporarily impaired arterial perfusion on the vulnerable biliary tree remains unclear as the OrganOx metra device provides supraphysiologic oxygenation of the portal venous blood (portal vein oxygen saturation is equivalent to arterial oxygenation). In agreement with this, Nasralla et al. found no biliary complications in patients with arterial reconstruction during NMP [10].

Second, pre-NMP reconstruction and not post-NMP distinguishes itself by its ability to perform simultaneous reperfusion, which has been shown to be associated with superior long-term outcome [11]. Third, pre-NMP reconstruction provides the option to modify the reconstruction if needed in a setting where the graft is non-perfused and freely positionable, i.e., during the back-table preparation. Although this did not occur in the current series, any form of problem arising from technical errors such as kinking or stenosis could have been corrected before beginning vascular anastomosis in the recipient to vascular reconstruction. Fourth, pre-NMP reconstruction of all vessels (irrespective of being an accessory or replaced artery) ensures a reliable graft viability assessment during NMP. Although some authors have suggested that aHA can be ligated in case of an adequate back-flow [12], we strongly believe that the reconstruction of all arteries is technically feasible and should, therefore, be done to truly ensure a perfectly perfused graft during NMP and in the recipient, thereby minimizing post-operative complications [13].

There are numerous practical lessons derived from our study: even complex reconstructions (e.g., Hiatt’s type IV with an absent CHA) are technically feasible and suitable prior to NMP. In addition, no arterial or venous autograft or allograft, or vascular prostheses were needed for reconstruction. However, meticulous hemostasis must be employed to avoid bleeding of the reconstruction during the red blood cell-based perfusion in the absence of platelets and clotting factors. Moreover, it is difficult to visually control the patency of a reconstructed artery, as the OrganOx metra device provides supraphysiologic oxygenated portal venous blood. Thus, even in a case of a closed artery, the respective lobe remains homogeneously marbled and appears visually intact. Therefore, it is advocated to use Doppler US and flow measurements in case of doubt.

Some surgeons advocate using only the celiac trunk including the LGA when faced with an aLHA, thereby avoiding the need for reconstruction, especially when the aLHA is rather small. However, we strongly advocate the reconstruction of these vessels (e.g., up to the origin of the GDA or RGA) to omit any vessel redundancy. The length of the reconstructed vessels should be kept to an absolute minimum to avoid kinking during NMP and in the recipient thereafter. With respect to this, the frequently described reconstruction onto the SMA was completely avoided by carrying out anastomoses only to the GDA branch or distal to it. Therefore, we routinely used the RGA and have not encountered a scenario in which this vessel was absent or too small. Additionally, in our experience, even very small accessory arteries are safe for reconstruction and NMP allows us to further evaluate their patency.

While others have declined grafts with aberrant arterial anatomy for NMP or to avoid difficult reconstruction, we, on the contrary, emphasize on the utilization of NMP in cases of complex arterial reconstruction. By employing NMP, one can test the reconstruction under physiological flow conditions, thereby shifting potential reconstruction hazards (such as bleeding, stenosis, kinking, and need for refashioning) from the recipient to the perfusion device. This is especially true for small accessory arteries. In addition, the absence of platelets and clotting factors during NMP seems to guarantee sufficient hemostasis after graft reperfusion in the recipient. It is important to state that we advocate our approach of pre-NMP reconstruction for all grafts, including marginal grafts, despite their known vulnerability to CIT. In our cohort, a total of four grafts (44.4%) were from extended criteria donors: one donor had a donor risk index (DRI) > 2, two liver grafts revealed a macrovesicular steatosis ≥ 30% and one graft had a CIT ≥ 10 h. However, even for marginal grafts for which reduction of CIT is critical, we believe that the above-mentioned benefits of pre-NMP reconstruction outweigh the additional CIT as a reconstruction, even a complex one, can be completed in under 30 min.

## Conclusion

Although our approach inevitably prolongs CIT by 10–30 min, it reduces the surgical difficulty of in vivo reconstruction and eliminates inadequate arterial graft perfusion during NMP. Since clinicians are frequently confronted with aberrant HAs, our data (although derived from only a small number of cases) demonstrates that even complex arterial reconstructions are technically feasible and no contraindication for successful NMP and subsequent transplantation.

## Data Availability

All the data and material necessary have been included in the study.
